# ArraySearch: A Web-Based Genomic Search Engine

**DOI:** 10.1155/2012/650842

**Published:** 2012-03-04

**Authors:** Tyler J. Wilson, Steven X. Ge

**Affiliations:** Department of Mathematics and Statistics, South Dakota State University, P.O. Box 2220, Brookings, SD 57007, USA

## Abstract

Recent advances in microarray technologies have resulted in a flood of genomics data. This large body of accumulated data could be used as a knowledge base to help researchers interpret new experimental data. ArraySearch finds statistical correlations between newly observed gene expression profiles and the huge source of well-characterized expression signatures deposited in the public domain. A search query of a list of genes will return experiments on which the genes are significantly up- or downregulated collectively. Searches can also be conducted using gene expression signatures from new experiments. This resource will empower biological researchers with a statistical method to explore expression data from their own research by comparing it with expression signatures from a large public archive.

## 1. Introduction

In recent years, there has been a massive influx of data from DNA microarray experiments into publicly available repositories such as the National Center for Biotechnology Information (NCBI). Many of these datasets represent genomewide expression profiles generated from experiments investigating controlled perturbations on molecular pathways, responses to chemical stimuli, responses to pathogen infections, or mutant behavior when certain genes are made inoperative. The largest repository of gene expression data is NCBI's Gene Expression Omnibus (GEO). As of July 1, 2011, GEO contains the expression profiles of 587,214 biological samples from different organisms. It is important to take advantage of this large body of data for the purpose of new genomics studies.

A number of sites and tools have become available that attempt to utilize the data in ways that will guide future research. Although GEO is primarily designed for information storage and downloading, GEO profiles provide methods for querying the expression profiles of genes (see http://www.ncbi.nlm.nih.gov/geoprofiles).

The Nottingham Arabidopsis Stock Center microarray service (NASCArrays) [1] also provides a number of useful data mining tools for exploring a large collection of gene expression data. The Two Gene Scatter Plot tool allows a user to visualize gene expression over all microarray slides for two genes as a scatter plot. The Spot History tool allows a user to see patterns of gene expression over all slides for specific genes.

Genevestigator [2] provides online tools to access large and well-annotated datasets of curated microarray samples. The meta-profile analysis function of Genevestigator summarizes gene expression data across many samples according to the biological context of the sample. Each gene signal in a meta-profile (in contrast to a normal expression profile) is the average of gene expression levels across samples sharing the same biological context (anatomy, development, stimulus, and mutation). Genevestigator also provides a useful method for single or biclustering of gene expression values based upon sample conditions, anatomy, genotype, or developmental stage.

Other sites, such as the Arabidopsis Information Resource Center (TAIR) [3] and plexDB.org [4], provide standard analytical tools such as gene expression graphs across samples for genes of interest, hierarchical and partitional clustering analysis, and the display of heat maps for genes across samples.

The web service Oncomine (http://www.oncomine.org/) created by Rhodes et al. [5, 6] provides an interface to a collection of cancer-related gene expression profiles and allows users to search a gene's expression across the entire collection. This tool has enabled researchers to make new discoveries, such as finding a common cancer signature defined by groups of genes that are consistently overexpressed in all cancers [7]. A newer version of the Oncomine site allows for the analysis of transcription factor binding motifs and has been instrumental in the identification of hundreds of gene regulatory programs associated with cancer [8].

The connectivity map (C-map) database collects the expression signatures of small molecule drugs [9]. C-map contains expression profiles of cell lines before and after the administration of hundreds of different drugs. It can be used to query an expression signature against these expression profiles to identify connections among small molecules sharing a common mechanism of action.

ArrayExpress [10] is a database for high-throughput functional genomics data for a number of different species. The database is comprised of two parts—the ArrayExpress repository which is a public data store of microarray data; and the ArrayExpress data warehouse, which is a collection of gene expression profiles taken from the repository. Gene expression profiles can be searched by gene names and properties, such as gene ontology terms, and can be visualized using the web interface: http://www.ebi.ac.uk/gxa/.

Although Oncomine, Genevestigator, and ArrayExpress provide facilities for searching through a database of gene expression profiles, they do not provide the ability for a researcher to submit queries with expression profiles from their own experiments and find contrasts highly correlated with their search query. The purpose of the ArraySearch website (http://ArraySearch.org/) is to leverage the existing large repository of genomics data by using it as a knowledge base for interpreting new data. ArraySearch achieves this goal by calculating statistical correlations between publicly available genomic data, which is well-characterized, and new experimental data.  Table 1 displays a comparison of features between ArraySearch, Genevestigator, plexDB, Oncomine, and ArrayExpress.

## 2. Materials and Methods

### 2.1. Downloading, Parsing, and Normalizing the Genomics Expression Data

Gene expression datasets from 160 experiments were downloaded from NCBI (http://www.ncbi.nlm.nih.gov/gds). Of these, 89 were then parsed and normalized using the Bioconductor package for R [11] to build the database ArraySearch uses. This is a multipart process, and the steps are summarized below for a single experiment.

1. Each experiment in the GEO Omnibus is referenced by an accession number. This number is used by the GEOquery library in bioconductor to download the raw data files for each sample in the experiment (referred to by their extensions as CEL files) from the NCBI ftp site. The files are then decompressed.
2. The gcrma package for R (also in bioconductor) is then used to process the raw data files for each sample in the experiment. This is done separately for each experiment. A group definition file is manually created which records which samples are control samples and which samples are experimental. For example, experiment GSE10928 contains 4 samples: GSM277232 … GSM277235. GSM27732 and GSM27733 are control sample replicates, while GSM277234 and GSM277235 are treatment samples. The treatment samples are placed in one group, and the control samples are placed in another group. The fold change values for the contrasts and the *P* values calculated using a paired *t*-test are then calculated and stored in separate tables.
3. The fold change (contrast) for each gene *g* in the genome is calculated as follows:
  (1) $$\text{fold}\left(g\right)=\log_{2}\left[\frac{\overset{̅}{g_{\text{treated}}}}{\overset{̅}{g_{\text{control}}}}\right],$$
  where
  (2) $$\begin{array}{c} \overset{̅}{g_{\text{treated}}}=\;\text{average}\;\text{gene}\;\text{expression}\;\text{for}\;\text{treated}\;\text{samples},\\ \overset{̅}{g_{\text{control}}}=\;\text{average}\;\text{gene}\;\text{expression}\;\text{for}\;\text{control}\;\text{samples}, \end{array}$$
4. A Bayesian *t*-test [12, 13] is used to compute the *P* values for each gene. Low *P* values are of interest because they inform us that the gene was upregulated or downregulated for this experiment. The fold change table reveals the direction of regulation.

### 2.2. The Technology behind ArraySearch

The ArraySearch website is composed of two parts: a front-end web interface created using the PHP scripting language and a back-end database created using MySQL. It was designed to be deployed on computers using Linux and running the Apache HTTP web server. The heat maps and bar graphs that ArraySearch generates for the different user queries were created using extensive modifications to a graphing library called pChart (http://pchart.sourceforge.net/) [14]. 

## 3. Results and Discussion

We downloaded gene expression datasets from 160 microarray experiments from NCBI (http://www.ncbi.nlm.nih.gov/gds). Only experiments using the Affymetrix ATH1 array were selected. Out of these experiments, only those with a minimum of two control and two treated arrays were considered for further analysis, resulting in 89 experiments. The samples within each experiment were divided into groups, and the groups were annotated with descriptions (e.g., WT root versus root: Sol2 mutant). A paired Bayesian *t*-test using the Cyber-T R script (http://cybert.ics.uci.edu/) [12, 13] was applied to the gene expression values in the control/treated samples. Small *P* values indicate there is a statistically significance between the gene expression values for the control relative to the treated values. The *P* values were stored in the *P* value table. The experiments were then manually curated, and the fold change values (contrasts) for each experiment were calculated. This resulted in 256 different contrasts.  Table 2 lists the experiments stored in the ArraySearch database, as well as the number of contrasts from each experiment. 

**Figure 1:**
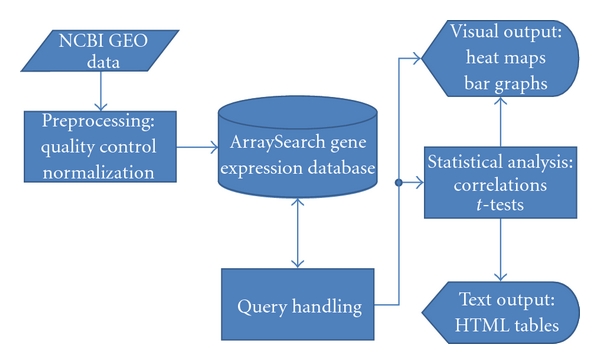
A flowchart indicating the various modules of the ArraySearch website and how they interact with each other.

The fold change is a measure of how much gene *g* is up/downregulated in the treated samples relative to the control samples. The details of this process are described for one experiment in Section 2. In Figure 1, a flowchart shows the various modules involved in creating the ArraySearch website and the flow of information between one module and the next.

### 3.1. The ArraySearch Database

**Figure 2:**
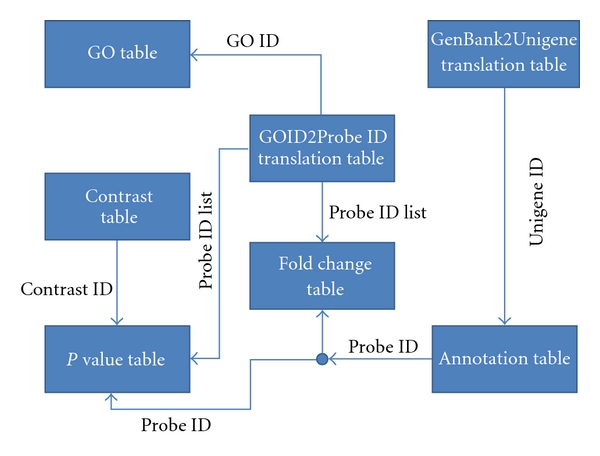
The structure of the ArraySearch database.

The structure of the database is shown in Figure 2. The ArraySearch site uses the database in two different ways:

1. When searching by expression signature, finds contrasts which are highly correlated with the expression profile the user entered,
2. When searching single/multiple gene, finds contrasts which are significantly up- or downregulated for the gene or list of genes entered.

The GO table in Figure 2 contains information about gene ontologies. Each gene ontology is referenced by a GOID number (e.g., GOID 15886 refers to “heme transport”). All genes in the tables are referenced by their Affymetrix probe ids, and the GOID2Probe ID table takes GOIDs and associates them with a list of probe IDs that correspond to genes that are actively expressed under the GOID. The GenBank2Unigene table is another translation table used to convert GenBank IDs into Unigene IDs. The annotation table contains information and aliases for all genes in the ATH1 array and is used to convert the Unigene IDs coming from the GenBank2Unigene table to their respective Affymetrix probe IDs. 

The contrast table contains information about each contrast in the database (study title, number of samples in the control and in the experiment, the study design, etc.). The *P* value table contains the *P* values for all of the contrasts in the database for every probe ID. 

The most important table in the ArraySearch database is the expression signature table. This table contains the fold change values for every gene on every contrast within the database.

### 3.2. Overview of the Search Functions

Four search functions are provided through the web interface:

1. Expression Profile Search finds experiments correlated with an expression signature;
2. Gene Ontology Search finds expression profiles for genes active in a gene ontological (GO) category search term (e.g., “defense response”);
3. Gene Title Search finds expression profiles for single genes;
4. GenBank Search returns expression profiles for a list of genes specified by their GenBank IDs.

Gene expression data currently come from one species: *Arabidopsis thaliana*. In the future, expression data from other species will be searchable.

### 3.3. Using the Expression Profile Search

As an example, a researcher is analyzing the results of a microarray experiment on *Arabidopsis*, investigating the effect of salt stress on gene expression along the radial axis of the root. In this experiment, three replicates of *Arabidopsis* root cells (from the columella root cap) were treated with 140 mM (millimolars) of NaCl. For the control, three replicates of *Arabidopsis* columella root cells were left untreated. The fold change value for each gene is calculated. Sorting the fold change values for all of the genes and looking at the top 10 most up-/downregulated genes gives the expression signature in Table 3.

A researcher wants to know other microarray experiments in which this pattern of up-/downregulated genes occurs. ArraySearch can answer this question. Entering this expression signature as an input query returns a list of contrasts highly correlated with the signature (see Table 4).

The actual search returns a list of the top 30 most highly correlated contrasts, along with summary information and links to NCBI where more information about the samples and the experiments can be obtained. The search results displayed in Table 4 show that the same genes in the input query exhibit similar expression patterns when ethylene and auxin interactions in the roots of *Arabidopsis* seedlings are triggered.

**Figure 3:**
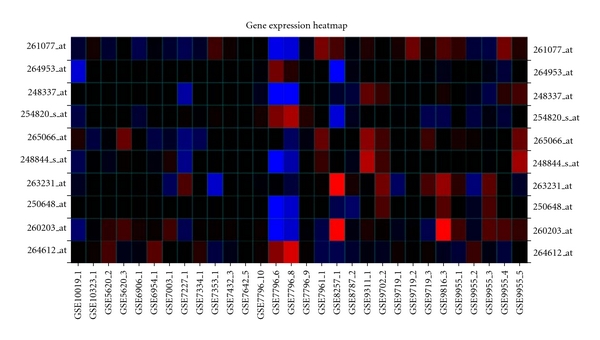
A heat map generated by the expression profile search function. Red cells indicate that the gene is upregulated on a contrast, and blue cells indicate that the gene is downregulated.

A heat map is also returned to the user. The color of each cell in the heat map is based on the expression value for that particular gene-sample pair. If a cell is bluish in color, then the gene is downregulated for that sample. If the cell is reddish in color, then the gene is upregulated for that sample (see Figure 3).

A researcher can also enter the list of probe IDs without the expression values, for example, 260130_s_at, 254889_at, 266353_at, 250500_at, 253667_at, 259813_at, 260668_at, 251065_at, 253024_at, 267121_at, 247333_at, and 252882_at. For this input, a two-tailed *t*-test is computed using the expression values for each gene across all contrasts in the database. All contrasts with *P*-values less than *α* = 0.05 are returned to the user, along with the *t*-statistic used to infer whether the genes as a group were down- or upregulated in an experiment. Summary information about each experiment, as well as links to NCBI, is also provided (see Table 5). Because this query involves multiple comparisons, the possibility of a number of false positives arising is a concern. To handle this, FDR *q*-values are also calculated for each contrast based on the method outlined in Benjamini and Hochberg [15].

### 3.4. Gene Ontology Search

The Gene Ontology Search allows users to find genes active for specific ontologies. To use this search, a gene ontological term such as “defense response” is entered. ArraySearch returns a list of Genome Ontology (GO) IDs along with their GO categories, which contain the search term. Searching for “defense response” returns Table 6. 

**Figure 4:**
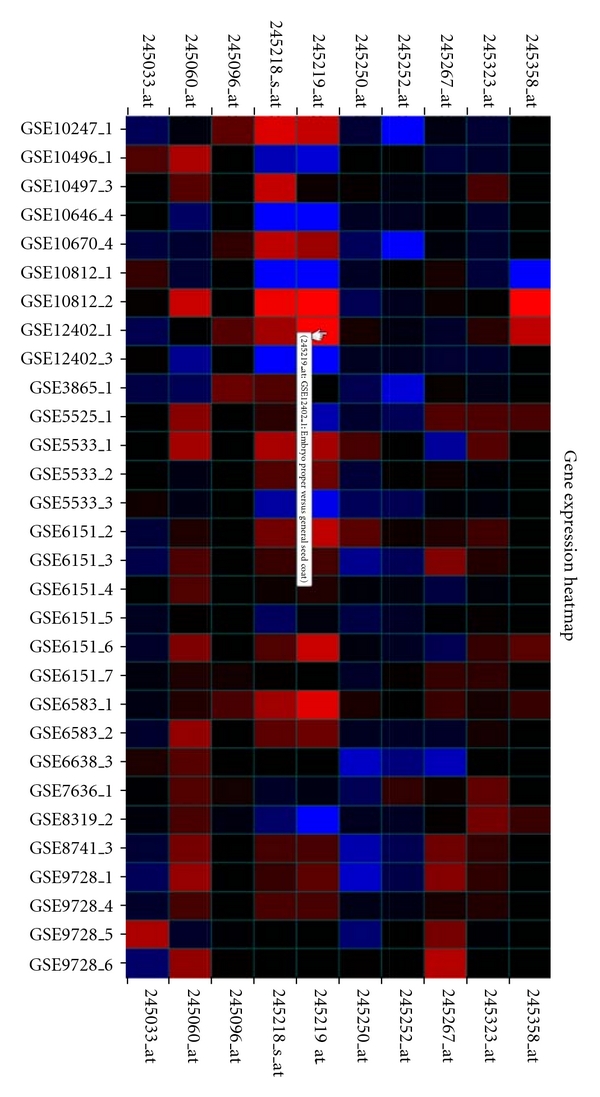
A heat map returned by the gene ontology search function. Red cells indicate that the gene is upregulated on a contrast, and blue cells indicate that the gene is downregulated. This heat map displays the 10 most active genes and their expression levels on the 30 most active contrasts returned by ArraySearch.

Each GO ID maps to one or more genes (denoted by their Affymetrix probe IDs) actively expressed in their respective ontologies. The output is displayed in a heat map (Figure 4), identical to the expression profile search. The heat map is 10 × 30, representing the expression values for the 10 most active genes and the 30 most active contrasts.

### 3.5. GenBank Search

**Figure 5:**
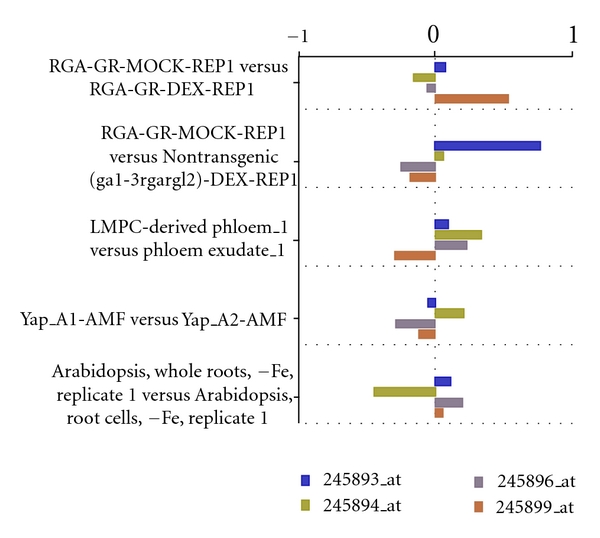
GenBank search results. Only the expression profiles for the first five experiments are shown above.

GenBank is the NIH DNA sequence database. It is a collection of all publicly released DNA sequences. Researchers often use GenBank IDs to refer to genes of interest; however, internally, ArraySearch refers to all genes by their Affymetrix probe IDs. This search function takes as input a tab-delimited list of one or more genes referred to by their GenBank Ids, for example, BP799393, DR371397, ES108934, and ES014211. It returns the expression profile for the gene set as a bar graph across all samples (see Figure 5).

### 3.6. Gene Title Search

The Gene Title Search function searches the ArraySearch database for all genes entered in the search term title. For example, a search for all genes with “Cold” in the title would return the list shown in Table 7 (which has been truncated slightly).

By default, this search returns a heat map identical to the gene ontology search function. That is, it displays the heat map showing the expression values of the ten most active genes over the thirty most active experimental samples in the ArraySearch database. Provided the number of genes returned by this search is reasonable (less than ten genes), a user may also elect to display a bar graph in addition to the heat map.

## 4. Conclusions

Genes within an organism seldom act individually, but work in concert with other genes in a complex network of interaction. Often the same group of genes behaves similarly in the presence of related biological stimuli. The major purpose of the ArraySearch website is to act as a genomic search engine that returns expression signature sets from different experiments when provided with an expression profile query of gene identifiers and corresponding fold change values. We hope this resource will empower biological researchers to explore expression data from their own research by comparing it alongside expression signatures from a large public archive.

## Figures and Tables

**Table 1:** A comparison of genomic search engine features.

	ArrayExpress	Oncomine	plexDB	Genevestigator	ArraySearch
[1] Website	http://www.ebi.ac.uk/gxa/	http://oncomine.org/	http://www.plexdb.org/	https://www.genevestigator.com/	http://ArraySearch.org/
[2] Microarray data source	ArrayExpress	ArrayExpress, GEO, SMD	AtGenExpress, NASCArrays, TAIR, datasets uploaded by plexDB curators	AtGenExpress, NCBI GEO, TAIR, ArrayExpress, NASCArrays, other sources …	NCBI GEO
[3] Species	*Arabidopsis*+others	*Human*	*Arabidopsis*+others	*Arabidopsis*+others	*Arabidopsis*
[4] statistical analysis					
[4.1] Query gene correlation with contrasts	No	No	No	No	Yes
[4.2] Query gene correlation by gene ontology	No	No	No	No	Yes
[4.4] User-defined expression profile query	No	No	No	No	Yes
[4.5] Correlation between query gene/samples	No	No	No	No	Yes
[5] Graphical output of gene coexpression	Heatmap	Scatter plot	Line graph/hierarchical Clustering	Line Graph	Heatmap/bargraph
[6] Output: coexpression scores between query genes	No	No	Yes	Yes	No
[7] References	Parkinson et al. [10]	Rhodes et al. [5]	Wise et al. [4]	Hruz et al. [2]	

**Table 2:** The experiments within the ArraySearch database.

GSE accession	Number of contrasts	Study title
GSE10019	2	Identification of RGA downstream genes by using steroid-inducible system
GSE10247	1	Transcriptome analysis of the Arabidopsis phloem
GSE10323	1	Testing Arabidopsis for the presence of arbuscular mycorrhizal signalling pathways
GSE10496	1	Expression analysis of the effect of protoplasting and FACS sorting in roots exposed to iron deficiency (-Fe)
GSE10497	4	Expression analysis of root developmental zones after iron deficiency (-Fe) treatment
GSE10502	6	Time course expression analysis of the iron deficiency (-Fe) response in Arabidopsis roots
GSE10509	4	EXECUTER1- and EXECUTER2-dependent transfer of stress signals from the plastid to the nucleus of Arabidopsis thaliana
GSE10522	10	Expression data of Arabidopsis thaliana rosettes during chilling
GSE10568	2	camta3 mutant versus wild-type microarray analysis
GSE10646	4	BTH treated mkk1, mkk2, and mkk1/2 knockout mutant
GSE10670	4	Global expression profiling of wild-type and transgenic Arabidopsis plants in response to water stress
GSE10719	1	Response of Arabidopsis cell culture to phytoprostane A1
GSE10732	4	Identification of TGA-regulated genes in response to phytoprostane A1 and OPDA
GSE10749	5	Response of Arabidopsis cell culture to cyclopentenone oxylipins
GSE10812	3	Expression data from thylakoidal ascorbate peroxidase overexpressor Arabidopsis thaliana (Col) rosette leaves
GSE10928	1	Arabidopsis wild-type versus vip3
GSE11119	1	SOL2 mutation affect gene expression at root apex
GSE11216	2	Brassinazole treatment of arf2 and wild-type dark-grown seedlings
GSE11558	5	transcript profiling of the adaptive response to decreases in oxygen concentration in the roots of Arabidopsis plants
GSE11594	1	Expression data from dark-grown Arabidopsis wild-type (Wt, col-o) and pif1-2 (At2g20810, Salk_072677) mutant seedlings
GSE11852	2	Effect of uniconazole on wt and pkl mutant germinating seeds
GSE12029	1	NFYA5, a CCAAT binding transcription factor important for drought resistance in Arabidopsis
GSE12137	1	LEAFY COTYLEDON1 is a key regulator of fatty acid biosynthesis in Arabidopsis thaliana
GSE12401	1	Transcript abundance data from seedlings of wild-type Ws and ged1 (greening after extended darkness 1) mutant
GSE12402	6	Expression data from Arabidopsis seed compartments at the preglobular stage
GSE3865	1	CSN4-1 mutant analysis
GSE3959	3	RNA changes induced by LEAFY COTYLEDON2 activity in Arabidopsis seedlings
GSE4113	3	Arabidopsis plants with altered levels of alternative oxidase
GSE4662	1	STA1, a stress-upregulated nuclear protein, is required for pre-mRNA splicing, mRNA turnover, and stress tolerance
GSE4733	8	Transcriptional regulators of stamen development in Arabidopsis identified by transcriptional profiling
GSE5465	2	Gene expression in wild-type and transgenic plants overexpressing rice topoisomerase6 genes
GSE5525	5	Transcriptome changes of Arabidopsis during pathogen and insect attack
GSE5526	3	Transcriptional programs of early reproductive stages in Arabidopsis
GSE5533	3	Tissue type arrays of Columbia-0
GSE5534	3	Response to cold, plate-grown plants
GSE5535	3	Response to cold, soil-grown plants
GSE5536	1	Response to CBF2 expression
GSE5611	1	Differential gene expression patterns in the phosphate-deficient mutant, pho 1
GSE5620	16	AtGenExpress: stress treatments (control plants)
GSE5641	1	Peroxisomal mdh mutant
GSE5699	1	AtGenExpress: ARR21C overexpression
GSE5710	1	Dark-induced gene expression in sfr6
GSE5712	1	Transcriptome analysis of ARRESTED DEVELOPMENT 3 mutant.
GSE5726	2	Seedling transcriptome affected by Norflurazon-induced photobleaching of chloroplasts
GSE5735	1	Identification of core genes regulating plant programmed cell death (PCD)
GSE5806	2	Identification of differentially expressed genes in brm-101 and syd-2 mutants
GSE6024	2	eif3h/WT polysome loading
GSE6025	1	eif3h/WT transcript level
GSE6151	9	The mechanisms involved in the interplay between dormancy and secondary growth in Arabidopsis
GSE6161	3	Differential gene expression patterns in Arabidopsis mutants lacking the K+ channels, akt1, cngc1, and cngc4.
GSE6166	1	Genes affected by hog1 alleviation of CHS silencing
GSE6583	2	Genomewide transcriptome analysis of Arabidopsis and siz1-3 response to drought stress
GSE6638	4	Expression data of germinating ahg1, ahg3, and WT seedling in the presence of ABA
GSE6696	2	Transcriptome analyses show changes in gene expression to accompany pollen germination and tube growth in Arabidopsis
GSE6788	1	Expression data of an albino mutant DS 13–2198-1
GSE6812	1	Gene expression in wild-type and transgenic plants overexpressing rice OsTOP6A1 gene
GSE6826	2	Identification of candidate Arabidillo target genes in Arabidopsis
GSE6827	1	Comparison of transcriptional profiles between sni1 and wild type
GSE6906	2	Rhythmic growth explained by coincidence between internal and external cues
GSE6954	2	Identification of AGL24 downstream genes by using XVE inducible system
GSE7003	1	Experiment to identify downstream targets of Arabidopsis REVOLUTA (HDZIPIII) transcription factor.
GSE7227	3	microRNA160-resistant AUXIN RESPONSE FACTOR10 (mARF10) germinating seeds
GSE7334	1	Microarray analysis of Arabidopsis genome Response to aluminum stress
GSE7353	2	Early GA response genes in Arabidopsis thaliana
GSE7432	4	Ethylene and auxin interactions in the roots of Arabidopsis seedlings
GSE7636	1	Expression analysis of the effect of protoplasting and FACS sorting in roots
GSE7639	3	Expression analysis of root developmental zones after treatment with salt
GSE7641	6	Expression analysis of root cell types after treatment with salt
GSE7642	5	Time course expression analysis of the salt stress response in Arabidopsis roots
GSE7796	10	Phenotypic diversity and altered environmental plasticity in Arabidopsis thaliana with reduced HSP90 levels
GSE7961	1	affy_aba_ath1-Characterization of aba3-1 suppressor mutants
GSE8257	1	Identification of KIN10-target genes in Arabidopsis mesophyll cells
GSE8279	2	Transgenerational stability of the Arabidopsis epigenome is coordinated by CG methylation
GSE8319	2	An LysM receptor-like kinase mediates chitin perception and fungal resistance in Arabidopsis
GSE8739	1	Early gibberellin responses in Arabidopsis
GSE8741	3	DELLA protein direct targets in Arabidopsis
GSE8745	1	Low R : FR treatment at 16 and 22 degrees
GSE8787	4	Expression analysis of the salt stress response in Arabidopsis mutants with defects in hair patterning
GSE9148	2	Expression data of 10-day-old wild-type and chl1-5 plants exposed to 25 mM nitrate for 0 h or 0.5 h
GSE9201	4	Identification of genes responding to the activity of the Arabidopsis cytochrome P450 KLUH/CYP78A5
GSE9311	2	Gene expression in roots and shoots of plants grown on selenate
GSE9408	1	Identification of putative Arabidopsis DEMETER target genes by GeneChip analysis
GSE9605	3	Target genes of AGAMOUS during early flower development in Arabidopsis
GSE9702	2	Identification of putative targets of AP3/PI
GSE9719	6	Dynamics of mRNA abundance and translation in response to short and prolonged hypoxia and reoxygenation
GSE9728	6	COP9 signalosome (csn) mutant analysis
GSE9816	3	Gene expression profiling of sav mutants
GSE9955	5	MILDEW-INDUCED LESIONS 4 encodes a novel regulator of the salicylic acid defense response
GSE9957	3	Expression profiling of the plant cell wall mutants: pmr5, pmr6, and pmr5 pmr6 double mutants

**Table 3:** The expression signature for the top 10 largest absolute fold change values.

Probe ID	Fold change
264612_at	−7.454
260203_at	−7.389
250648_at	−6.247
263231_at	−6.239
248844_s_at	5.912
265066_at	5.652
254820_s_at	5.64
248337_at	−5.624
264953_at	−5.6
261077_at	−5.547

**Table 4:** The output of expression profile search returns a list of other experiments highly correlated with the query expression signature. This is an abbreviated table. The full search results contain more information.

Contrast ID	Pearson correlation	Study title
GSE7641_2	1.00000000	Expression analysis of root cell-types after treatment with salt
GSE9816_1	0.97571633	Gene expression profiling of sav mutants
GSE9728_3	0.96537382	COP9 signalosome (csn) mutant analysis

**Table 5:** Expression profile search results for a probe list query. The *t*-statistic indicates whether the query list is up- or downregulated on an experiment. This is an abbreviated version of the actual search results.

Contrast ID	*P* value	FDR *q*-value	t-statistic	Study title
GSE10928_1	0.00088	0.04449	4.5172	Arabidopsis wild-type versus vip3
GSE11119_1	0.00104	0.04449	−4.4115	SOL2 mutation affects gene expression at root apex
GSE12029_1	0.00529	0.10564	−3.4642	NFYA5, a CCAAT binding transcription factor important for drought resistance in Arabidopsis
⋮	⋮	⋮	⋮	⋮

**Table 6:** Gene ontology categories returned by searching for “defense response.” Each GO ID has an associated set of genes actively expressed for that ontology.

GO identifier	GO category
6952	Defense response inferred from reviewed computational analysis
42742	Defense response to bacterium inferred from mutant phenotype
9816	Defense response to bacterium, incompatible interaction inferred from mutant phenotype
9870	Defense response signalling pathway, resistance gene-dependent inferred from mutant phenotype

**Table 7:** Gene list generated by gene title search.

Probe ID	Gene title
246251_at	Cold acclimation protein homolog…
246481_s_at	Cold and ABA inducible protein kin1
247700_at	RNA-binding protein—like cold-inducible RNA-binding…
249966_at	putative protein COLD-INDUCIBLE RNA-BINDING PROTEIN
⋮	⋮
